# Long Non-Coding RNAs Identified as Hub Genes by Weighted Gene Co-Expression Network Analysis in *Schistosoma mansoni* Following Incubation with *Bothrops* Snake Venoms

**DOI:** 10.3390/ijms27115027

**Published:** 2026-06-02

**Authors:** Marina Zenga-Carrenho, Agatha Fischer-Carvalho, Tereza Cristina Taveira-Barbosa, Pedro Jardim Poli, Vilaça Guimarães-Oliveira, Alison Felipe Alencar Chaves, Solange M. T. Serrano, Ana Carolina Tahira, Sergio Verjovski-Almeida, Murilo Sena Amaral

**Affiliations:** 1 Laboratório de Ciclo Celular, Instituto Butantan, São Paulo 05503-900, SP, Brazil; 2 Programa de Pós-Graduação Interunidades em Biotecnologia, Universidade de São Paulo, São Paulo 05508-000, SP, Brazil; 3 Laboratório de Toxinologia Aplicada, Center of Toxins, Immune-Response, and Cell Signaling (CeTICS), Butantan Institute, São Paulo 05503-900, SP, Brazil; 4 Departamento de Bioquímica, Instituto de Química, Universidade de São Paulo, São Paulo 05508-000, SP, Brazil

**Keywords:** schistosomiasis, *Schistosoma mansoni*, *Bothrops* venoms, lncRNAs, RNA-Seq, WGCNA

## Abstract

Emerging tolerance of *Schistosoma mansoni* to praziquantel, the only drug available for schistosomiasis treatment, highlights the need for new therapeutic targets. Snake venoms contain pharmacologically active proteins and peptides that can decrease the viability of *S. mansoni* worms in vitro. Long non-coding RNAs (lncRNAs) play important roles in *S. mansoni* and are promising new therapeutic targets. However, new candidates still need to be identified, as only four *S. mansoni* lncRNAs have been functionally characterized to date. Therefore, we investigated lncRNA expression changes in *S. mansoni* following incubation with *Bothrops* venoms. Adult worms were incubated with eight venoms at a sublethal dose, and phenotypic parameters were evaluated. RNA-Seq was conducted on worms incubated with *Bothrops jararacussu* or *Bothrops moojeni* venoms, followed by Weighted Gene Co-expression Network Analysis for each sex. *B. moojeni* venom reduced all phenotypic measurements, while *B. jararacussu* reduced oviposition. Both venoms altered global gene expression, including lncRNAs. Females showed two lncRNA hub genes in two venom-associated co-expression modules, while males showed 61 lncRNA hub genes in nine venom-associated modules. RT-qPCR validated six out of seven selected hub lncRNAs in male worms. These results reveal the involvement of lncRNAs in *S. mansoni* gene expression modulation induced by *Bothrops* venoms and point to lncRNAs that should be prioritized in future functional studies, such as *SmLINC121220-IBu*, *SmLINC152105-IBu* and *SmLNCA123831-IBu*.

## 1. Introduction

Schistosomiasis is a neglected disease caused by trematodes of the genus *Schistosoma*, with 240 million people requiring preventive chemotherapy and about 700 million people living in at-risk areas [1,2]. The disease has two main forms: urogenital schistosomiasis, caused by *Schistosoma haematobium*, and intestinal schistosomiasis, mainly caused by *Schistosoma japonicum* and *Schistosoma mansoni* [3]. Praziquantel (PZQ) is the only drug currently indicated for the treatment of schistosomiasis. However, there are reports of parasite tolerance to the drug [4]. In addition, PZQ is not effective against immature forms of the parasite (schistosomula) [5,6]. Therefore, the search for new pharmacological targets and novel antischistosomal compounds is necessary.

Currently, most candidate compounds are plant-derived, which have been extensively studied for their antischistosomal properties [7]. In contrast, animal-derived compounds have received comparatively less attention, as reviewed by our group in [8], despite being a major focus of drug discovery for various other therapeutic applications [9,10]. To date, few studies have reported the antischistosomal activity of animal natural products, although with promising results [11,12,13,14,15,16,17,18]. In this context, snakes of the genus *Bothrops* are of major medical importance in Central and South America, as they are responsible for the highest rates of envenomation in the region [19]. *Bothrops* snake venoms have complex composition, being rich in components with pharmacological and biotechnological potential, such as metalloproteases and serine proteases, phospholipases A2, L-amino acid oxidases, 5′-nucleotidases, hyaluronidase, and C-type lectins [20,21], which disrupt multiple molecular targets in the victim. Bites from *Bothrops* snakes trigger intense local reactions, typically involving severe pain, inflammation, blistering, and tissue necrosis. Beyond the bite site, the venom’s systemic impact can cause low blood pressure, blood clotting disturbance, and bleeding gums. In the most critical instances, this can escalate to circulatory collapse and kidney failure [22,23].

Long non-coding RNAs (lncRNAs) are transcripts longer than 200 nucleotides with low or no protein-coding potential [24,25,26], proposed to be used in new therapeutic strategies against several diseases [27,28]. In mammals, lncRNAs are known to play numerous roles in organism homeostasis, such as gene regulation and drug resistance [29,30,31,32]. In *S. mansoni*, lncRNAs were first described in 2011 [33] and have since been identified across different parasite life-cycle stages [34,35,36,37,38]. Recently, lncRNAs were shown to be essential for *S. mansoni* adult worm homeostasis, survival and fertility [39,40] and, therefore, proposed as putative new therapeutic targets against schistosomiasis [40]. LncRNAs were also shown to be sensitive to stress in *S. mansoni*, with their expression levels modulated by drugs such as PZQ in vivo [41] and the ribonucleoside analog 5-azacytidine (5-AzaC) in vitro [42]. Since only four out of thousands of lncRNAs have been silenced and functionally evaluated in *S. mansoni* so far [39,40], new candidate lncRNAs still need to be identified.

Thus, this study aimed to identify lncRNA expression patterns in *S. mansoni* adult worms under stress induced by incubation with *Bothrops* snake venoms. In addition to pointing to the molecular mechanisms by which *Bothrops* venoms induce parasite death and potential target genes, this work aimed to open new perspectives through a broader evaluation of *S. mansoni* gene expression following venom incubation using RNA-Seq and Weighted Gene Co-expression Network Analysis (WGCNA). Thus, by identifying a set of *S. mansoni* genes affected by *Bothrops* venoms, this study indicates lncRNAs that should be prioritized in future functional studies focusing on the development of new lncRNA-based therapeutic alternatives against schistosomiasis.

## 2. Results

### 2.1. Bothrops moojeni In Vitro Incubation Reduces Pairing, Adhesion, Motility, Viability, and Oviposition of Schistosoma mansoni Adult Worms

**Figure 1:**
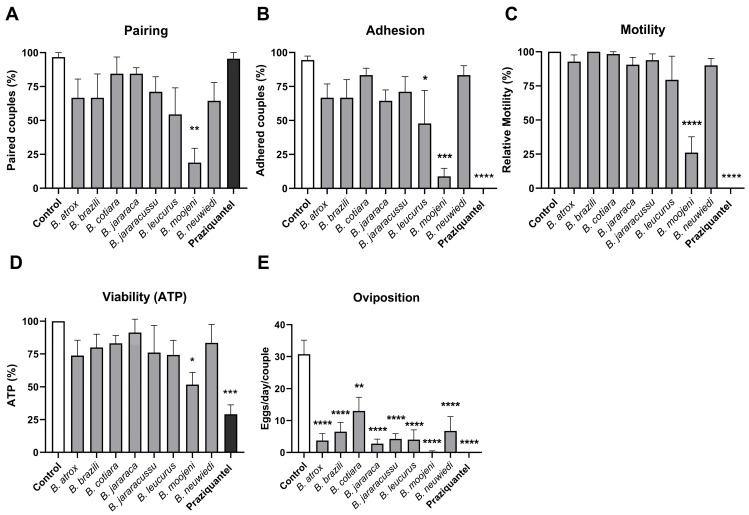
Evaluation of adult *Schistosoma mansoni* viability following in vitro incubation with venoms from eight different snake species of the genus *Bothrops*. Adult worms were incubated for 24 h with each of the indicated venoms separately at a concentration of 25 µg/mL or with praziquantel (1.5 µg/mL) as a positive control. (**A**) Percentage of paired worm couples after incubation. (**B**) Percentage of worm couples attached to the plate after incubation. (**C**) Percentage reduction in worm motility levels relative to control worms. (**D**) Quantification of ATP levels. The mean of two technical replicates from each of four biological replicates per condition was used. (**E**) Number of eggs laid per worm pair over 24 h. Means ± SEM of three (**A**–**C**) and four (**D**,**E**) biological replicates are shown. A one-way ANOVA with Dunnett’s post-hoc correction test was applied. (*) = *p* < 0.05, (**) = *p* < 0.01, (***) = *p* < 0.001, and (****) = *p* < 0.0001.

To assess possible differential expression of lncRNAs upon incubation of *S. mansoni* adult couples with *Bothrops* venoms, we first screened the antischistosomal potential of eight venoms obtained from the snakes *Bothrops atrox*, *Bothrops brazili*, *Bothrops cotiara*, *Bothrops jararaca*, *Bothrops jararacussu*, *Bothrops leucurus*, *Bothrops moojeni*, and *Bothrops neuwiedi* using a sublethal dose (25 µg/mL) to select venoms for further RNA-Seq experiments. Only the venom of *B. moojeni* significantly reduced pairing of adult worms, with only 18.9% remaining paired following exposure to venom (Figure 1A). Regarding attachment to the plate, venoms from *B. moojeni* and *B. leucurus* led to a significant reduction in worm attachment to 8.9% and 47.7%, respectively (Figure 1B). Concerning motility and viability, only the *B. moojeni* venom caused a significant decrease to 26% and 52% in the relative motility index and ATP levels of adult worms, respectively (Figure 1C,D). All eight venoms tested significantly reduced oviposition (Figure 1E), with *B. moojeni* venom causing almost complete inhibition of oviposition. Considering that the *B. moojeni* venom was the only one that reduced all phenotypic features measured, and that the *B. jararacussu* venom reduced oviposition and is a venom well known for its richness in molecules with pharmacological potential [43,44,45,46], we performed RNA-Seq with adult samples incubated with either *B. moojeni* or *B. jararacussu* venom. Supplementary Table S1 shows the list of all *S. mansoni* transcripts detected in the RNA-Seq, along with their TPM (transcripts per million) values.

### 2.2. S. mansoni Males Show a Higher Number of Differentially Expressed (DE) Genes than Females in the RNA-Seq

**Figure 2:**
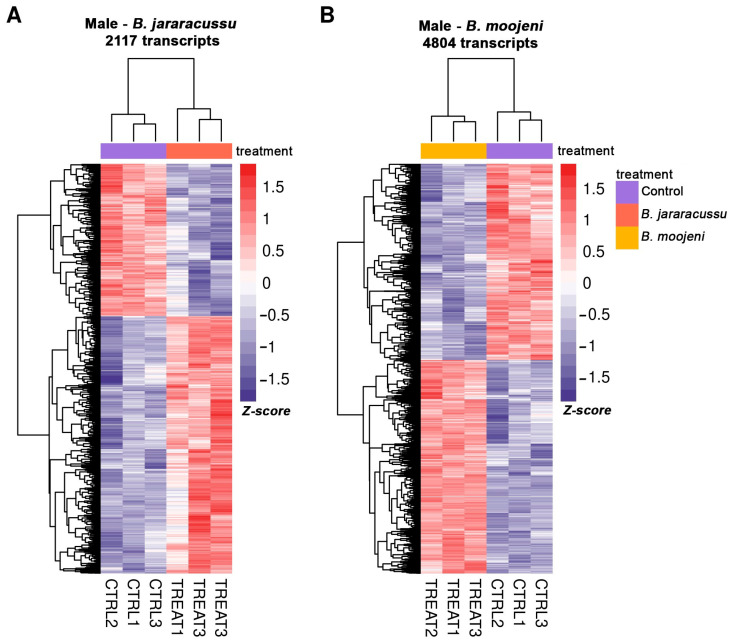
Heatmaps of differentially expressed (DE) transcripts in *Schistosoma mansoni* male samples incubated with a sublethal dose (25 µg/mL) of *Bothrops jararacussu* (**A**) or *Bothrops moojeni* (**B**) venom compared with their respective controls. Gene expression levels were measured by RNA-Seq and are shown as Z-scores, which are the number of standard deviations below (downregulated, light blue) or above (upregulated, red) the mean expression value among treated and control samples for each gene; Z-score color scale indicated on the right. Each gene is in a horizontal line; replicate samples are in the columns. TREAT = sample incubated with *Bothrops* venom. CTRL = negative control sample. Statistical significance: FDR ≤ 5%.

Incubation of *S. mansoni* adult worm couples with a sublethal dose (25 µg/mL) of both *B. jararacussu* or *B. moojeni* venoms significantly changed the global profile of gene expression in males. RNA-Seq analysis showed that males incubated with *B. jararacussu* venom had 2117 significantly (FDR ≤ 5%) differentially expressed genes (DEGs) (Figure 2A), while males incubated with *B. moojeni* showed the highest number of significantly (FDR ≤ 5%) DEGs, 4804, compared with the controls (Figure 2B).

**Figure 3:**
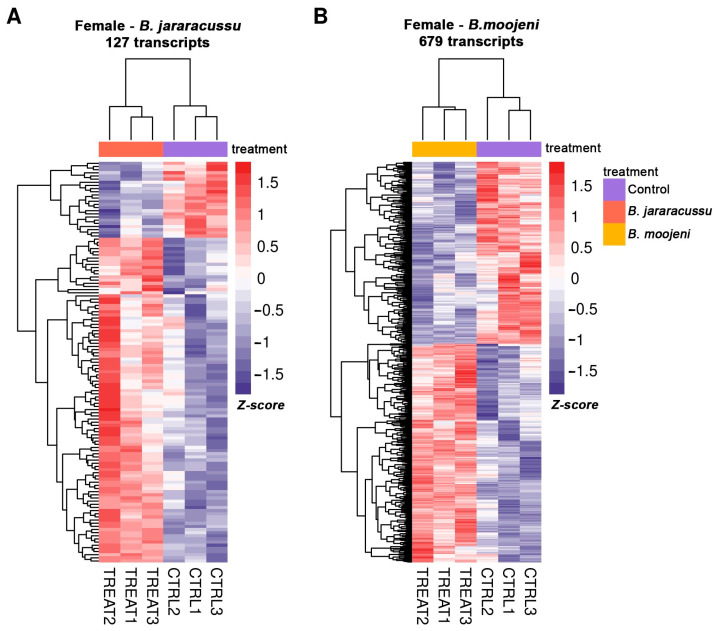
Heatmaps of differentially expressed transcripts in *Schistosoma mansoni* female samples incubated with a sublethal dose (25 µg/mL) of *Bothrops jararacussu* (**A**) or *Bothrops moojeni* (**B**) venom compared with their respective controls. Gene expression levels were measured by RNA-Seq and are shown as Z-scores, which are the number of standard deviations below (downregulated, light blue) or above (upregulated, red) the mean expression value among treated and control samples for each gene; Z-score color scale indicated on the right. Each gene is in a horizontal line; replicate samples are in the columns. TREAT = sample incubated with *Bothrops* venom. CTRL = negative control sample. Statistical significance: FDR ≤ 10%.

Meanwhile, females exposed to the same concentration (25 µg/mL) of either of the two venoms showed a considerably lower number of significantly (FDR ≤ 5%) DEGs compared with males. To account for the pronounced sexual distinction in the gene expression response, we applied a more permissive threshold for females (FDR ≤ 10%) compared with the FDR used for males; of note, at a strict FDR ≤ 5%, females yielded insufficient DEGs (0 to 116) for downstream analyses, unlike the much more robust response observed in males. Thus, a total of 127 significantly (FDR ≤ 10%) DEGs were detected (Figure 3A) following females’ exposure to *B. jararacussu* venom, while exposure to *B. moojeni* venom led to 679 significantly (FDR ≤ 10%) DEGs (Figure 3B), when compared with the respective controls. Lists of all significantly DE protein-coding genes and lncRNAs from Figure 2 and Figure 3 are given in Supplementary Tables S2–S5.

**Figure 4:**
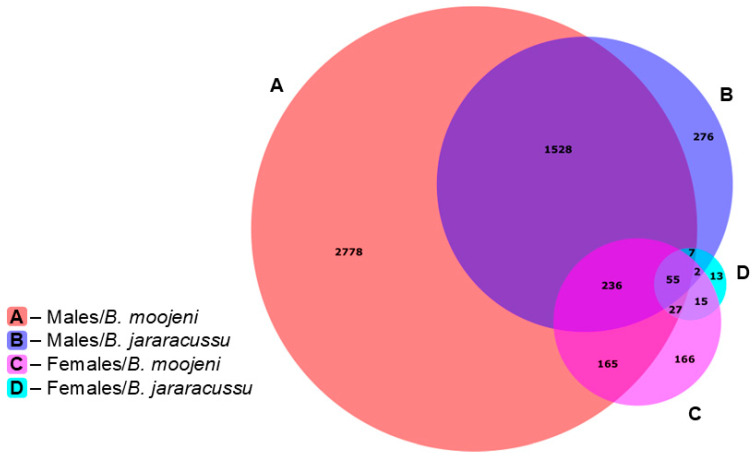
Venn Diagram showing the number of significantly differentially expressed genes (DEGs) in the intersections of data from male or female *S. mansoni* worms exposed separately to either *B. moojeni* or *B. jararacussu* venoms. (**A**) Red: number of DEGs found in *S. mansoni* males exposed to *B. moojeni* venom. (**B**) Blue: number of DEGs found in *S. mansoni* males exposed to *B. jararacussu* venom. (**C**) Fuchsia: number of DEGs found in *S. mansoni* females exposed to *B. moojeni* venom. (**D**) Cyan: number of DEGs found in *S. mansoni* females exposed to *B. jararacussu* venom. Not shown in the diagram are the following intersections: A ∩ D = 8 genes; B ∩ C = 13 genes. Diagram drawn with the online tool https://www.deepvenn.com/.

In addition, when comparing the overlap of DEGs between parasites exposed to either *B. moojeni* or *B. jararacussu* venoms, 86% of DEGs affected in males by *B. jararacussu* venom are also detected as DEGs under incubation of males with *B. moojeni* venom (Figure 4A,B). Similarly, in females, 78% of DEGs affected by *B. jararacussu* venom are also DEGs following *B. moojeni* venom incubation (Figure 4C,D). These findings suggest that within each sex, the two venoms affect similar pathways on the parasites; nevertheless, *B. moojeni* venom affects a considerably larger number of genes in both sexes than *B. jararacussu* venom.

### 2.3. LncRNAs Are Differentially Expressed (DE) in S. mansoni Males and Females Incubated with B. jararacussu or B. moojeni Venoms

Among the DEGs detected in male or female worms exposed to both venoms, there was a relevant proportion of significantly DE lncRNAs. Except for females incubated with *B. jararacussu*, in which 6% of the total DEGs were lncRNA genes, approximately 9–10% of the total DEGs of all other treatment groups were lncRNAs (Supplementary Table S6). Of interest, most of the significantly DE lncRNAs are long intergenic non-coding RNAs (lincRNAs, 62.5% to 71.6% of total DE lncRNAs), followed by long antisense non-coding RNAs (lncaRNAs, 26.9% to 37.5% of total DE lncRNAs), and long sense non-coding RNAs (lncsRNAs, 0% to 4.4% of total DE lncRNAs) (Supplementary Tables S2–S5).

### 2.4. S. mansoni Males Show More lncRNAs Hubs of Modules Significantly Associated with Venom Incubation than Females

Next, we used WGCNA to look for co-expression between lncRNAs and protein-coding genes, attempting to infer which functional pathways in the parasites were significantly affected by the genome-wide expression reprogramming induced by venom exposure, and which functional roles the lincRNAs may have. In the WGCNA of female samples exposed to *B. jararacussu* or *B. moojeni* venoms, a total of 49 co-expression modules were identified, with an average of 353 genes per module. The largest module was turquoise, containing 1506 transcripts, whereas the smallest module was plum2, containing 150 transcripts. Among the identified modules, nine of them showed a statistically significant association with treatment with one or both venoms (*p*-value < 0.05), and these modules, along with modules comprising DE lncRNAs, are shown in Supplementary Table S7. Furthermore, in females, exposure to *B. moojeni* venom had a greater impact on gene expression than exposure to *B. jararacussu* venom, both in terms of the number of DEGs identified and in their significant association with modules in each analysis (Supplementary Table S7).

**Figure 5:**
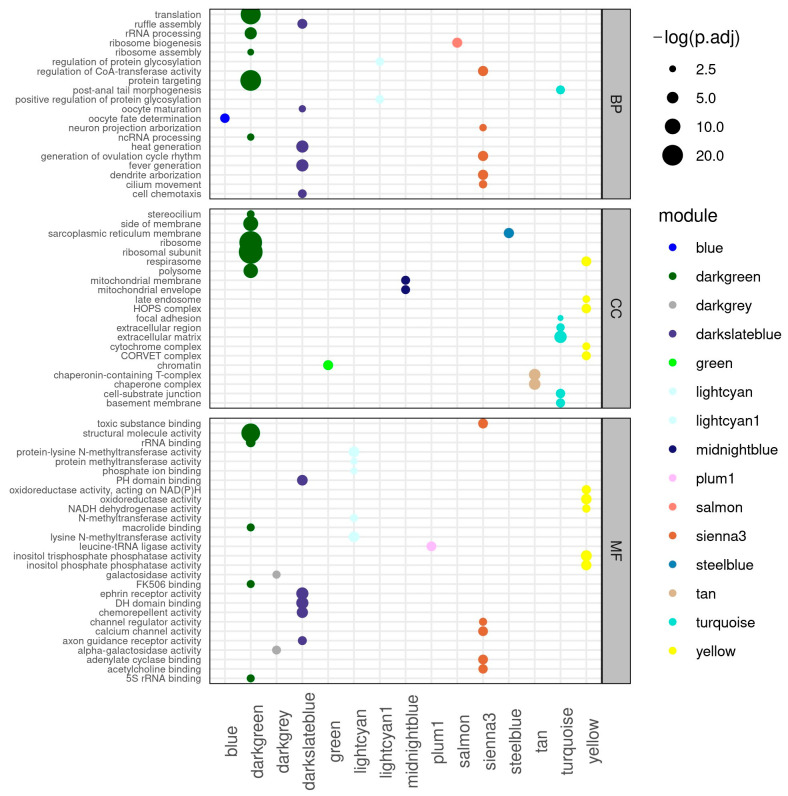
Gene Ontology (GO) terms enrichment within the WGCNA modules from *S. mansoni* female samples. Modules are shown in the columns, and each row represents an enriched GO term (FDR ≤ 10%) in each of the three categories, namely Biological Process (BP), Cellular Component (CC), and Molecular Function (MF), as indicated on the gray column at right. The significance of enrichment is proportional to the size of the dots. Colors of the dots correspond to the color names of modules. The top five enriched GO terms per module are displayed.

To identify the modules that were significantly associated with specific biological functions in females, we performed a clusterProfiler analysis and found that 15 modules showed significantly enriched Gene Ontology (GO) terms across the three GO categories, namely Biological Process (BP), Cellular Component (CC), and Molecular Function (MF) (Figure 5 and Supplementary Table S8). The darkslateblue module presented the highest number of significantly enriched GO terms, totaling 138, with 131 GO terms in the BP category and 7 in the MF category; the top five enriched GO terms in the BP category are oocyte maturation, ruffle assembly, cell chemotaxis, heat generation and fever generation.

Moreover, 105 transcripts were identified as both hub genes and DEGs in at least one of the venom treatments in female samples. Of these, 25 transcripts were hubs and DEGs in the *B. jararacussu* treatment across five modules, and 103 transcripts were hubs and DEGs in the *B. moojeni* treatment across 19 modules, with the brown module containing the highest number of hub DEGs. Since it was possible to design RT-qPCR primers for only one DE lncRNA hub out of the two present in these modules, we did not proceed to RT-qPCR validation of any target in female samples.

Interestingly, in the WGCNA of male parasite samples exposed to *B. jararacussu* or *B. moojeni* venoms, a total of 21 modules were identified, each one containing an average of 790 genes. The largest module was turquoise, containing 4288 transcripts, whereas the smallest module was dark red, containing 223 transcripts. Among the identified modules, ten of them showed a significant association with one or both venom treatments (*p*-value < 0.05) (Supplementary Table S9). In males, as in females, exposure to *B. moojeni* venom had a greater impact on gene expression than exposure to *B. jararacussu* venom, both in terms of the number of DEGs identified and their significant association with modules in each analysis (Supplementary Table S9).

In the *B. jararacussu* treatment, five modules were significantly associated with the venom exposure: one positively associated (turquoise) and four negatively associated (green, greenyellow, red, and royalblue). In three of these modules (green, red, and turquoise), the enrichment with DEGs is significantly associated with the modules (Supplementary Table S9). In contrast, in the *B. moojeni* treatment, eight modules were significantly associated with the venom exposure: four positively associated and four negatively associated (Supplementary Table S9). In all these modules, except for brown and light yellow, the enrichment with DEGs is significantly associated with the modules. The turquoise module was enriched with the highest number of DEGs in both venom incubations: 1326 out of 2147 in the *B. jararacussu* treatment, and 1960 out of 5662 in the *B. moojeni* treatment (Supplementary Table S9). In the clusterProfiler analysis across the three GO categories (BP, CC, and MF) to identify modules significantly associated with specific biological functions, 11 modules presented enriched GO terms, with the brown module showing the highest number of significantly enriched GO terms (339) (Supplementary Table S10).

Furthermore, 1031 transcripts were identified as both hub genes and DEGs in at least one of the venom treatments. Of these, 519 transcripts were hubs and DEGs in the *B. jararacussu* treatment across 10 modules, and 985 transcripts were hubs and DEGs in the *B. moojeni* treatment across 11 modules. Based on this analysis, we selected and were able to design efficient primer pairs for RT-qPCR validation for seven DE hub lncRNAs in *S. mansoni* males: five lincRNAs (*SmLINC115582-IBu*, *SmLINC116522-IBu*, *SmLINC121220-IBu*, *SmLINC150454-IBu* and *SmLINC152105-IBu*), one lncaRNA (*SmLNCA123831-IBu*) and one lncsRNA (*SmLNCS129871-IBu*).

**Figure 6:**
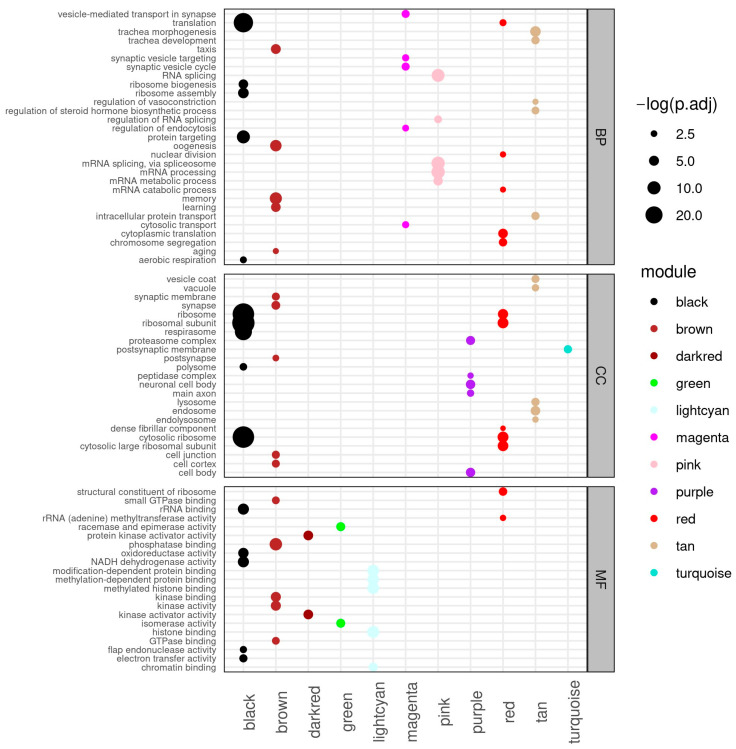
Gene Ontology GO terms enrichment within the WGCNA modules from *S. mansoni* male samples. Modules are shown in the columns, and each row represents an enriched GO term (FDR ≤ 10%), in each of the three categories, namely Biological Process (BP), Cellular Component (CC), and Molecular Function (MF), as indicated on the gray column at right. The significance of enrichment is proportional to the size of the dots. Colors of the dots correspond to the color names of modules. The top five enriched GO terms per module are displayed.

**Figure 7:**
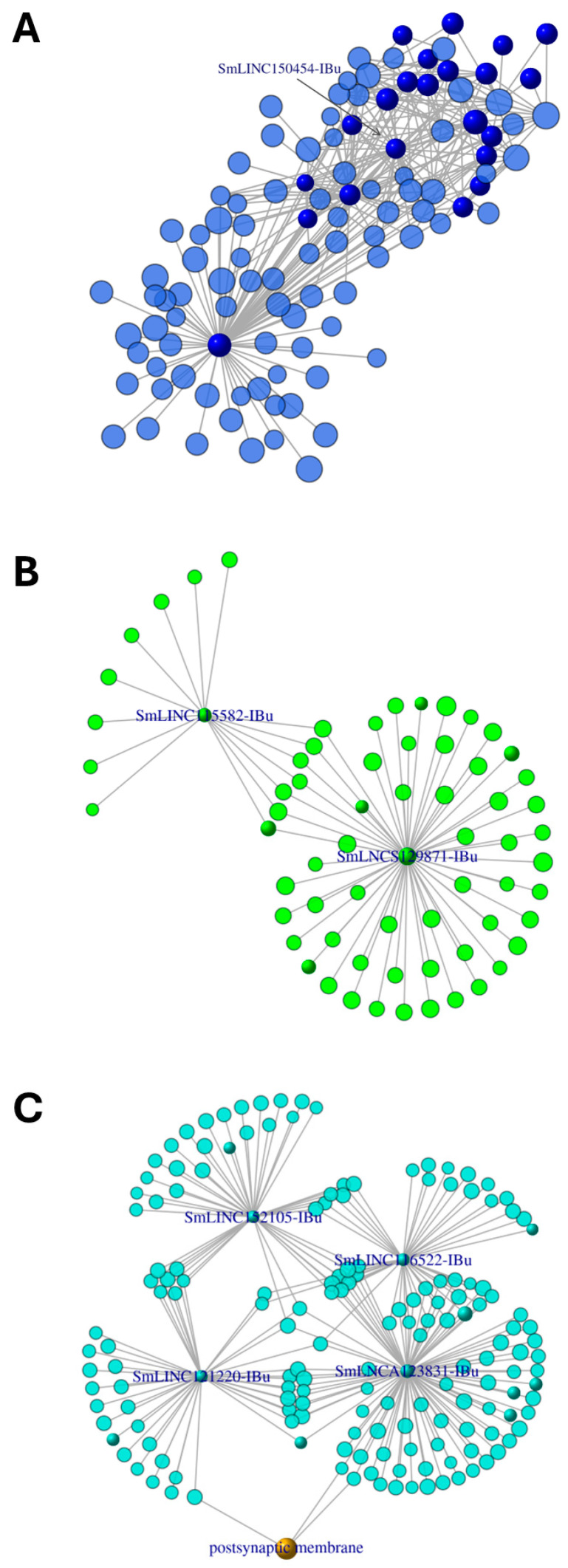
Co-expression networks of modules Blue (**A**), Green (**B**) and Turquoise (**C**) identified by Weighted Gene Co-expression Network Analysis (WGCNA), highlighting long non-coding RNAs (lncRNAs) selected for validation by RT-qPCR. Only hub lncRNAs from each module were retained (kME > 90th percentile). To further reduce network complexity, edges were filtered to include only correlations above the 75th percentile. Connections involving the selected lncRNAs of interest were highlighted within each module. Darker nodes represent lncRNAs, lighter nodes represent protein-coding genes, and yellow nodes indicate enriched GO terms.

Four of these selected hub lncRNAs (*SmLINC116522-IBu*, *SmLINC121220-IBu*, *SmLINC152105-IBu* and *SmLNCA123831-IBu*) belong to the turquoise module, while two belong to the green module (*SmLINC115582-IBu* and *SmLNCS129871-IBu*) and one (*SmLINC150454-IBu*) to the blue module (Supplementary Table S11). Blue module has no GO term enrichment, while the green module is enriched with MF GO terms related to isomerase activity, racemase and epimerase activity, and turquoise is enriched with the CC GO term related to postsynaptic membrane (Figure 6). Modules blue, green and turquoise co-expression networks are shown in Figure 7. While the blue module showed no significant GO enrichment (Figure 7A), the green module displayed enriched GO terms and two hub lncRNAs (*SmLINC115582-IBu* and *SmLNCS129871-IBu*) (Figure 7B). Notably, the turquoise module presented one enriched GO term (postsynaptic membrane) connected to three of the selected differentially expressed lncRNAs (Figure 7C), suggesting potential functional associations within this module.

### 2.5. Six lncRNA Hubs Were Validated by RT-qPCR in S. mansoni Male Samples Incubated with B. jararacussu or B. moojeni Venoms

**Figure 8:**
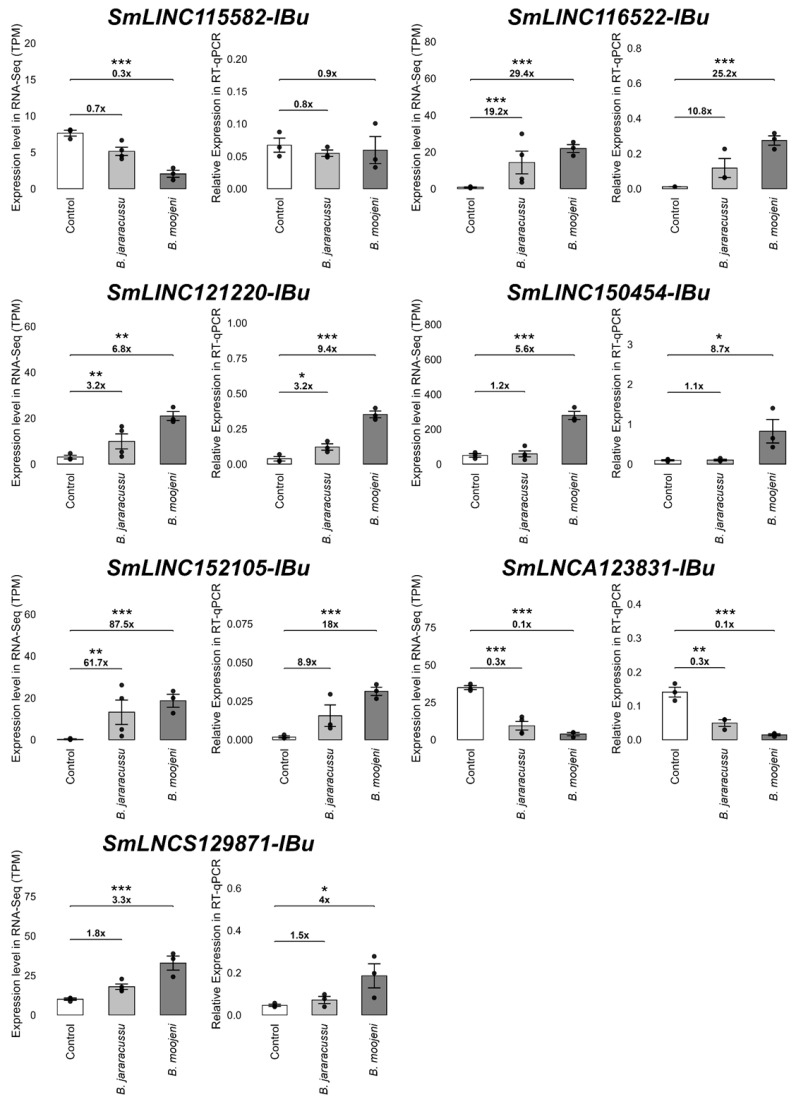
Expression level of selected lncRNAs in male *S. mansoni* samples incubated with *B. jararacussu* or *B. moojeni* venoms. For each selected lncRNA indicated at the top of the panel, the expression obtained by RNA-Seq (TPM, transcripts per million) (left three columns) or by RT-qPCR (right three columns) is shown for the control assay (left), for *B. jararacussu* exposure (middle) and for *B. moojeni* exposure (right). Relative gene expression by RT-qPCR was calculated using the delta Ct approach, considering each pair of primers’ efficiency and normalizing to the average of two selected reference genes (*Smp_136320.1* and *Smp_336360.1*). Means ± SEM of three biological replicates were used. Numbers above the horizontal lines across the columns indicate the fold-changes (FCs), which were calculated by dividing the treatment gene expression value by the control gene expression value in both RNA-Seq and RT-qPCR results. Student’s *t*-test between the treated sample group and its respective control group was applied assuming equal variances. (*) = *p* < 0.05, (**) = *p* < 0.01, (***) = *p* < 0.001.

Overall, the gene expression of the DE lncRNAs evaluated by RT-qPCR presented a similar pattern as that in the RNA-Seq analyses, with correlation coefficients higher than 0.84 (Supplementary Figure S1). In the RT-qPCR, parasites incubated with *B. moojeni* venom showed, as in the RNA-Seq, significantly higher expression of *SmLINC116522-IBu*, *SmLINC121220-IBu*, *SmLINC150454-IBu*, *SmLINC152105-IBu* and *SmLNCS129871-IBu*, and significantly lower expression of *SmLNCA123831-IBu* when compared with control samples (Figure 8). Meanwhile, male parasites incubated with *B. jararacussu* venom showed significantly higher expression of *SmLINC121220-IBu* and lower expression of *SmLNCA123831-IBu* compared with control samples (Figure 8).

We also selected, as a control, a lncRNA (*SmLINC143821-IBu*) that is not DE in male samples incubated with neither of the venoms. In the RT-qPCR, *SmLINC143821-IBu* showed a similar gene expression between control and incubated samples as in the RNA-Seq (Supplementary Figure S2). Thus, six DE hub-lncRNAs (*SmLINC116522-IBu*, *SmLINC121220-IBu*, *SmLINC150454-IBu*, *SmLINC152105-IBu, SmLNCA123831-IBu* and *SmLNCS129871-IBu*) and one non-DE lncRNA (*SmLINC143821-IBu*) had RNA-Seq results validated by RT-qPCR in samples of *S. mansoni* males incubated with *Bothrops* venoms.

## 3. Discussion

In this study, we showed that *Bothrops* snake venoms, such as *B. jararacussu* or *B. moojeni*, can phenotypically affect *S. mansoni* worms following in vitro incubation for 24 h with a sublethal dose of 25 µg/mL. Incubation with *B. moojeni* venom significantly reduced parasite pairing, adhesion, motility, viability, and oviposition, representing a suitable candidate for further studies aiming at investigating and isolating the venom molecules responsible for these observed effects. The phenotypical results are consistent with previous reports of in vivo and in vitro treatment targeting *S. mansoni* with *Cerastes cerastes* venom and in vivo administration of MjTX-II, a phospholipase A2 homolog obtained from *B. moojeni,* which led to reduced viability and oviposition, along with other phenotypical results [12,14,18]. Together with these previous findings, our results emphasize the little explored potential of snake venoms as a source of pharmacologically active molecules against parasites such as *S. mansoni* [8].

In addition to the phenotypical effects, incubation with *B. jararacussu* or *B. moojeni* venoms also led to molecular changes in both male and female *S. mansoni* worms. Male worms showed a higher number of DEGs than females after incubation with each of the venoms, showing that the gene expression effects of exposure to *Bothrops* venoms are higher in male than female worms. It has been shown that male *S. mansoni* are phenotypically more susceptible to in vitro incubation with some compounds, including PZQ [5], ginger (*Zinziber officinale*) extract [47], some alkylated diamines and amino alcohols [48], essential oil of *Ageratum conyzoides* [49], and *C. cerastes* venom [14]. Still, female worms are more susceptible to in vitro incubation with artesunate [50] and anti-malarial drugs [51], while there is no differential sensitivity between sexes upon incubation with (+)-Limonene Epoxide [52], *Plectranthus neochilus* [53] and *Piper cubeba* [49] essential oils. Thus, these differences in susceptibility between male and female *S. mansoni* may be associated with intrinsic sex-specific characteristics that lead to different levels of sensitivity depending on the mechanism of action of the compound.

The precise mechanisms of action of the venoms in schistosomes are unknown; considering the higher sensitivity of males to venom incubation, it is possible that these mechanisms may involve direct effects on the tegument and, consequently, the activation of surface receptors. This direct effect could explain the more pronounced molecular changes observed in males, as they have a larger contact surface with the environment than females. Meanwhile, male and female worms differ in their tegumental composition regarding molecules such as proteins, lipids and N-glycans [54,55,56]. Specifically, male lipid tegumental composition, with higher fatty acyl substituents and a higher number of double bonds compared with females, leads to a higher fluidity in male tegument compared with females [55]. Considering that *Bothrops* snake venom composition includes enzymes such as metalloproteases, serine proteases, and phospholipases [20,21], the difference in tegumental fluidity and composition regarding proteins, lipids and N-glycans may lead to a higher male sensitivity to the venoms. Furthermore, in adult *S. mansoni* worms, there is a larger number of male-biased than female-biased genes, i.e., there are more genes with significantly higher expression in males than in females [57]. In this context, male worms could show an enhanced gene expression regulation after exposure to *B. jararacussu* or *B. moojeni* venoms due to their higher sensitivity to the venom mechanism of action and/or because of their higher transcriptional plasticity, caused by male-biased genes, in response to stressful conditions.

In addition, enriched GO terms in male modules significantly associated with one or both venom incubation conditions (Supplementary Table S12) point to a systemic collapse involving basic cellular machinery (“cytoplasmic translation”; “nuclear division”; “chromosome segregation”), profound epigenetic reprogramming (“histone binding”; “methylated histone binding”; “chromatin binding”), and involvement of neuromuscular function and electrolyte balance (“postsynaptic membrane”; “voltage-gated potassium channel complex”; “ion channel complex”). In contrast, enriched GO terms in female modules significantly associated with venom incubation (Supplementary Table S13) indicate a more structural and restricted response, involving regulation of genes related to surface integrity (“extracellular matrix”; “integral component of plasma membrane”; “collagen-containing extracellular matrix”) and reproduction (“female germline ring canal”; “female germline ring canal inner rim”). Ultimately, this difference in molecular responses may reveal a more tightly regulated gene expression program in females, which is more resistant to venom-induced reprogramming than in males. These data suggest a molecular basis for the sex-specific susceptibility to venom, demonstrating a pervasive systemic collapse in the male compared with the more targeted structural response in the female.

In this study, 6–10% of the genes significantly DE upon incubation with either of the two venoms in male and female worms are lncRNAs. LncRNAs play essential roles in eukaryotes’ gene regulation [29,30] and are associated with drug resistance mechanisms [31], presenting a strong potential as therapeutic targets against parasites, including schistosomes [39,40,58]. In *S. mansoni*, lncRNAs are known to be essential for worm homeostasis and reproduction [39]. They are modulated upon parasite drug exposure to drugs, such as in the in vivo treatment with PZQ [41], the only currently indicated treatment for schistosomiasis, or in the in vitro incubation with 5-AzaC, an epigenetic drug [42]. Thus, investigating lncRNAs affected by *Bothrops* snake venom incubation may help to identify lncRNAs that are modulated in the parasites under stress, which can be tested in the future as putative pharmacological alternatives against schistosomiasis.

To date, only four lncRNAs have been functionally evaluated and silenced in *S. mansoni* [39,40]. *SmLINC156349-IBu* knockdown decreased *S. mansoni* viability and oviposition, and impaired parasite pairing, adhesion, and motility [40]. Meanwhile, *SmLINC101519-IBu*, *SmLINC110998-IBu* and *SmLINC175062-IBu* knockdown reduced parasite pairing, adhesion, motility and/or oviposition, while *SmLINC101519-IBu* silencing reduced worms’ viability [39]. These results show the role of lncRNAs in *S. mansoni* homeostasis and reproduction and highlight the potential of these RNAs as therapeutic targets while also emphasizing how much is yet to be discovered about lncRNAs in *S. mansoni*. While most of these previously studied lncRNAs are not DE in the context of this study, *SmLINC101519-IBu* is significantly DE in males incubated with *B. moojeni*, and some of them are part of modules significantly associated with venom incubation obtained in the WGCNA. *SmLINC101519-IBu* is part of the brown module in males and the black module in females, both significantly associated with *B. moojeni* venom incubation, while *SmLINC156349-IBu* is part of the green module in males, which is significantly associated with incubation with both *B. moojeni* and *B. jararacussu* venoms. This highlights that these lncRNAs are possibly involved in the molecular changes induced by the venoms and indicates that the exposure to venoms, especially in males, modulated pathways containing lncRNAs previously known to be essential for *S. mansoni* homeostasis and reproduction.

In this study, WGCNA pointed to lncRNAs that act as hubs in modules significantly associated with venom incubation, especially in male worms. These lncRNA hubs are likely to be involved in the molecular pathways affected by incubation with the venoms, possibly regulating transcriptionally or post-transcriptionally the expression of the other genes detected in the modules, including protein-coding genes. Validation by RT-qPCR showed that six out of the seven selected lncRNAs are significantly DE in *S. mansoni* male samples incubated with *B. moojeni* or *B. jararacussu* venoms. Therefore, this validation reinforces the potential of some of these selected lncRNAs as new candidates for further functional assays involving their knockdown or overexpression.

Among the selected lncRNAs, *SmLNCA123831-IBu* appears as one of the strongest candidates for further studies. This lncRNA is one of the most highly expressed transcripts in male *S. mansoni* out of the seven lncRNAs selected for validation, with an average TPM of 28.48 (Supplementary Figure S3) [59]. *SmLNCA123831-IBu* was downregulated in male samples incubated with both *B. jararacussu* and *B. moojeni* venoms, and this result was validated by RT-qPCR after incubation with both venoms. Meanwhile, this lncRNA is also downregulated in *S. mansoni* adult worm couples obtained from mice after treatment with a sub-lethal dose of PZQ at both four and seven weeks post-infection [41] and in females treated in vitro with 5-AzaC [42]. In the context of this study, *SmLNCA123831-IBu* is a hub of the turquoise module, significantly associated with incubation with both *B. jararacussu* and *B. moojeni* venoms in males. Together, these results suggest that *SmLNCA123831-IBu* may have a role in pathways downregulated in the parasite under stressful conditions, which are common among exposure to PZQ, to 5-AzaC and to the studied *Bothrops* venoms.

Furthermore, *SmLNCA123831-IBu* is an antisense lncRNA that overlaps the protein-coding gene *Smp_012380.1* (putative voltage-gated potassium channel, subunit β) at the genomic opposite strand. Antisense lncRNAs in eukaryotes can exert diverse biological and cellular roles by positively or negatively regulating the expression of nearby genes either *in cis* or *in trans* [60,61,62]. Thus, *SmLNCA123831-IBu* may act as a regulator of *Smp_012380.1* expression. This protein-coding gene is annotated as a putative subunit β of a voltage-gated potassium channel and is part of the WGCNA purple module in males. This module is significantly associated with incubation with *B. moojeni* venom and shows 25 enriched cellular component GO terms, including cell body, main axon, neuronal cell body, peptidase complex and proteasome complex (Figure 6). Meanwhile, the turquoise module in males, which contains *SmLNCA123831-IBu*, has one enriched GO term related to postsynaptic membrane. Interestingly, turquoise and purple modules are highly negatively correlated in the WGCNA (Supplementary Figure S4) and show enriched GO terms related to neural information transmission. Additionally, single-cell data (Supplementary Figure S5) point to *SmLNCA123831-IBu* enrichment in cell clusters of tegumental development (*meg1+* and *egc1+*) in males [63,64]. Tegumental membrane potential in adult male *S. mansoni* is known to be primarily dependent on the potassium gradient across the surface [65]. Therefore, we suggest that *SmLNCA123831-IBu* may interfere with potassium channel expression during worm tegumental development, being critical for parasite surface integrity and, therefore, for survival in human hosts. Considering its upregulation in PZQ, 5-AzaC and *Bothrops* venom exposures, *SmLNCA123831-IBu* shows up as a strong candidate for further functional studies and assays, such as in vitro and in vivo knockdown.

Other promising candidates pointed out by this study are *SmLINC150454-IBu* and *SmLINC121220-IBu*. In the RNA-Seq, *SmLINC150454-IBu* is upregulated in males incubated with *B. moojeni* venom and was validated by RT-qPCR. This lncRNA is also upregulated in adult worm couples obtained from mice treated with PZQ at seven weeks post-infection [41] and in females treated in vitro with 5-AzaC [42]. *SmLINC150454-IBu* has higher expression in *S. mansoni* schistosomula (Supplementary Figure S3) [59], although it shows high expression in male samples, with an average TPM of 24.52. This lncRNA also shows ubiquitous expression in single-cell expression data (Supplementary Figure S5) [63]. In humans, ubiquitously expressed lncRNAs were identified to serve universal housekeeping functions, being required for the maintenance of basal cellular functions [66]. Therefore, the ubiquitous and high expression of *SmLINC150454-IBu* in males, together with its upregulation after *Bothrops* venoms, PZQ and 5-AzaC treatment contexts, suggests a putative role of *SmLINC150454-IBu* in gene expression modulation of male *S. mansoni* under stressful contexts and highlights this lncRNA as a promising candidate for further investigations.

Notably, in the RNA-Seq, *SmLINC121220-IBu* was upregulated in male samples incubated with *B. moojeni* venom and was also validated by RT-qPCR. Despite not showing a high expression in male *S. mansoni* (TPM average of 4.8) (Supplementary Figure S3), *SmLINC121220-IBu* shows a considerable expression throughout several cell clusters in single-cell RNA-Seq data (Supplementary Figure S5), which could indicate a role in basal cellular functions [66]. In the present study, *SmLINC121220-IBu* is a hub in the turquoise module in males, and its expression is significantly correlated with protein-coding genes related to GO terms enrichment in the post-synaptic membrane (Figure 7). These results suggest a putative role of *SmLINC121220-IBu* in gene expression modulation upon *B. moojeni* venom incubation in male *S. mansoni*, possibly related to nervous information transmission across the parasite, making it worth further in-depth analyses. It is important to note that GO annotations in the genome of *S. mansoni* are less complete than in model organisms, which may have limited the identification of additional pathways affected by venom incubation in this study.

Altogether, these findings indicate that *Bothrops* snake venoms, especially *B. moojeni*, affect key biochemical pathways essential for parasite survival and therefore exhibit a strong potential for the development of pharmacologically active molecules against *S. mansoni.* This effectiveness stems from the biochemical complexity of snake venoms, which typically induce systemic shock and profound molecular disruptions in prey, comprising a multi-target mechanism that appears to be mirrored in the significant alterations observed in the treated parasites. Phenotypical and molecular results of this study highlight the importance of future studies testing venom fractions and investigating their pharmacologically active molecules against *S. mansoni*. Meanwhile, WGCNA showed that lncRNAs are not only modulated by *B. moojeni* or *B. jararacussu* venom incubation in male samples but are also hubs of modules significantly associated with treatment, i.e., they may play a role in gene expression modulation by venoms. Three out of the seven lncRNA hubs evaluated here (*SmLNCA123831-IBu*, *SmLINC150454-IBu* and *SmLINC121220-IBu*) are proposed as particularly promising candidates for further functional assays, including in vitro and in vivo silencing and whole-mount in situ hybridization [39,63]. Overall, this study presents the first results of large-scale molecular changes in *S. mansoni* adult worms following snake venom incubations and points to new *S. mansoni* lncRNAs that should be prioritized in future functional studies.

## 4. Materials and Methods

### 4.1. Parasite Material

The BH (Belo Horizonte) strain of *S. mansoni* is maintained in the intermediate host, the snail *Biomphalaria glabrata*, and in the definitive host, golden hamsters (*Mesocricetus auratus*). For the recovery of adult worms, 3–4-week-old hamsters were infected by exposure to a suspension containing approximately 200–250 *S. mansoni* cercariae [67]. After 49 days, adult *S. mansoni* worms were recovered by portal hepatic perfusion. The recovered adult parasites were maintained in BME medium (Vitrocell, Campinas, SP, Brazil) supplemented with 10% fetal bovine serum (Vitrocell, Campinas, SP, Brazil), 2% penicillin/streptomycin, and 0.5% gentamicin (Vitrocell, Campinas, SP, Brazil) at 37 °C and 5% CO_2_.

### 4.2. Parasite Incubation with Venoms

Adult *S. mansoni* worm couples were incubated with each *Bothrops* venom at a concentration of 25 µg/mL (sublethal dose) for 24 h in 12-well plates, with each well containing 2 mL of supplemented BME medium and 5 couples. Four biological replicates were performed, and 10–15 worm couples were used in each condition for each biological replicate. The venoms tested were obtained from the snakes *B. atrox*, *B. brazili*, *B. cotiara*, *B. jararaca*, *B. jararacussu*, *B. leucurus*, *B. moojeni*, and *B. neuwiedi*. The venoms were supplied in lyophilized form by the Strategic Center for Venoms and Antivenoms (NEVAS, Butantan Institute, São Paulo, Brazil). Each venom sample consisted of a pooled venom extracted from animals from different regions of Brazil. The samples were diluted in sterile PBS to a concentration of 1 mg/mL. Protein concentrations of each venom were determined using the BCA reagent (BCA Protein Assay Kit, Thermo Fisher Scientific, Waltham, MA, USA), with bovine serum albumin as a standard (Sigma-Aldrich, St. Louis, MO, USA). The samples were then aliquoted and stored at –80 °C until use. As a positive control, adult worms were incubated with PZQ at a concentration of 1.5 µg/mL. After incubation, worms were collected, washed in PBS, and stored in RNAlater (Thermo Fisher Scientific, Waltham, MA, USA) for RNA extraction.

### 4.3. Parasite Viability Evaluation

Using a light microscope (Olympus CKX41, Münster, Germany), adult worms were evaluated according to three parameters: pairing, attachment to the plate, and motility. To assess motility, the criteria described by Horiuchi et al. [68] were followed, in which three motility scores were established: score 3 (full-body movement), score 1.5 (partial movement or live but immobile worms), and score 0 (worms considered dead). For percentage calculation, the motility index (MI) was determined as MI=∑nNn/∑Nn, where Nn represents the number of worms with score n. Relative motility (RM) was calculated as (MIsample/MIcontrol)×100. Adult worm viability was determined by measuring ATP levels using the CellTiter-Glo kit (Promega, Madison, WI, USA), according to the manufacturer’s recommendations and as previously performed by our group [39,69]. In this assay, the CTG reagent, which generates a luminescent signal, was added to the previously treated and freshly lysed adult parasites, and luminescence was measured 15 min after reagent addition. The luminescence-based viability assay determines the amount of ATP present in the parasites, indicating the presence of cells with higher or lower metabolic activity. PBS was used as a negative control. The medium in which parasites were cultured was collected for egg counting. After medium collection, wells were washed with PBS to ensure complete collection of eggs. The medium was briefly centrifuged for egg deposition, the volume was adjusted to 1000 μL and 100 μL aliquots were taken for egg counting under a bright light with 4× magnification using an Olympus CKX41 inverted microscope (Münster, Germany). Counting was performed after in vitro incubation with venoms (or controls) for 24 h, and the number of eggs/couple/day of incubation was then estimated.

### 4.4. RNA Extraction and cDNA Synthesis

After adult worms’ collection and storage, worm couples were manually separated using tweezers and a stereomicroscope (Motic, Carlsbad, CA, USA), while maintaining parasites in a plate containing RNAlater (Thermo Fisher Scientific, Waltham, MA, USA). RNA extraction was performed separately for females and males. Total RNA extraction from the parasites was carried out using the RNeasy Micro kit (Qiagen, Hilden, Germany) in three biological replicates, according to the manufacturer’s instructions and as routinely performed by our group [39,41,42]. RNA purity was assessed by measuring RNA sample absorbance at 260, 280, and 230 nm. RNA integrity was verified by capillary electrophoresis using the 2100 Bioanalyzer (Agilent, Santa Clara, CA, USA) with the Agilent RNA 6000 Pico kit protocol. RNA concentration was determined using the Qubit 2.0 Fluorometer (Invitrogen, Waltham, MA, USA) and a NanoDrop spectrophotometer (Thermo Fisher Scientific, Waltham, MA, USA). RNA was extracted from control adult worms (incubated with PBS) and from worms incubated with *B. jararacussu* or *B. moojeni* venoms at a concentration of 25 µg/mL for 24 h, in three biological replicates. Synthesis of cDNA for RT-qPCR was performed using 200 ng of each adult worm sample and Superscript IV Kit (Thermo Fisher Scientific, Waltham, MA, USA), following the manufacturer’s instructions [39,41,42].

### 4.5. RNA-Seq

RNA-Seq was performed with samples from male and female worms (three biological replicates each) incubated with *B. jararacussu* or *B. moojeni* venom or samples incubated with PBS (negative controls). Strand-specific cDNA libraries were constructed from the extracted RNA samples. Library preparation was carried out by BGI Genomics (Shenzhen, China) using the “Strand-Specific Transcriptome Library Construction” protocol (DNBSEQ, SOP-SS-115), and library concentration and quality were assessed using the 2100 Bioanalyzer (Agilent). Libraries were pooled and sequenced (300 cycles, paired-end reads) on a DNBSEQ-G400 instrument (MGI Tech, Shenzhen, China). Sequencing data were deposited at the NCBI Sequence Read Archive (SRA) under the BioProject accession number PRJNA1442209.

RNA-Seq analyses to identify statistically significant differentially expressed genes (DEGs) were conducted following previously established and published protocols from our group [39,70]. In brief, read quality was assessed using FASTQC (v0.11.7), and adapter trimming and removal of low-quality reads were performed using FASTP (v0.20.0) [71]. RNA-Seq reads were mapped against the *Schistosoma mansoni* genome PRJEA36577 (v7), retrieved from WormBase (schistosoma_mansoni.PRJEA36577.WBPS14.genomic_softmasked.fa) with a transcriptome annotation previously published by our research group [34]. This transcriptome comprises 10,520 protein-coding genes and 16,583 lncRNAs annotated in version 7 of the *S. mansoni* genome (v7). Alignment was performed using the STAR algorithm (v2.7.0c) [72] with default parameters, modifying only the maximum number of multiple alignments per read (20×) and the maximum mismatch rate relative to read length (set to 0.3). Read counting was conducted using RSEM (v1.3.1) [73] with the same transcriptome previously published by our group [34]. Statistical analysis to identify DEGs was performed by comparing each venom-treated group with the control group using two distinct algorithms: (i) limma (v3.48.3) + voom (v3.48.3) [74] and (ii) edgeR (v3.34.1) + svaseq (v 3.40.0) [75,76]. Only genes identified as differentially expressed (DE) in both analyses were reported. DEGs are presented in an unsupervised hierarchical clustering analysis constructed using the pheatmap function (v1.0.12) in the R platform (v4.1.0) [77].

### 4.6. Weighted Gene Co-Expression Network Analysis

Weighted Gene Co-Expression Network Analysis (WGCNA, v1.70-3) aims to evaluate gene expression patterns to construct co-expression modules [78,79]. To build these modules, it is necessary to assume a scale-free topology, as described in Network Medicine [80], in which many genes have few connections while a few genes, referred to as network hubs, have many connections. To construct a scale-free network, the algorithm uses a soft-thresholding power (β), which is selected to maximize scale-free network properties. Thus, the adjacency matrix containing gene–gene correlations was raised to the selected power until scale-free topology was achieved (Supplementary Figure S6). For network construction, the selected power was 14 for females and 7 for male samples (Supplementary Figure S6). After this step, a Topological Overlap Matrix (TOM) was calculated, and distances were derived from the corresponding dissimilarity matrix. Genes with low dissimilarity distance were then grouped into the same module (Supplementary Figure S7). All networks were constructed as “unsigned,” meaning that both positive and negative correlations were considered in building the co-expression network. Herein, the analysis was performed using RNA-Seq data to guide the selection of central (hub) lncRNA genes for further validation by RT-qPCR, focusing on those showing altered expression in adult worms following incubation with *Bothrops* venoms. The analysis was conducted separately for *S. mansoni* males and females incubated with *B. jararacussu* or *B. moojeni* venoms and their respective negative controls.

A total of nine samples per sex were analyzed, comprising: three samples of females and three samples of males incubated with *B. jararacussu* venom, three samples of females and three samples of males incubated with *B. moojeni* venom, and three control female and three control male samples. In the first step, transcripts with a median expression equal to zero were filtered out. Next, genes were normalized using the variance stabilizing transformation (VST), and the coefficient of variation (CV) was calculated. The 1% of genes with the lowest CV were removed, resulting in 18,028 transcripts for females and 17,999 transcripts for males. Genes with low variation across samples tend to be less meaningful in biological contexts, and filtering them reduces noise. At this stage, one control female sample was excluded due to an outlier clustering pattern. After module identification, module preservation analysis was performed in both datasets with 100 permutation rounds and varying sample sizes (5 to 9) to test the robustness of each module; all of them resulted in a score (Z-summary preservation) > 10, which reflects topological preservation even at the smaller sample sizes [81]. After verifying the robustness of each module, a linear model statistical analysis was performed in R [77] to determine which modules were significantly associated with venom treatment, using the group as a categorical independent variable. The response variable consisted of the eigengenes of each module, which summarize the overall expression pattern of the genes within each module. Additionally, a χ^2^ test was performed to assess whether there was an association between modules and DEGs. Only results with a *p*-value ≤ 0.05 and in which the observed number of genes exceeded the expected number were considered significant.

To determine whether modules were associated with specific biological functions, enrichment analysis was conducted using clusterProfiler (v4.0.5) [82] in R (v4.1.0) [77]. First, genes were annotated using the eggNOG 5.0 (v 5.0.2) database [83], and enrichment analysis was then performed with clusterProfiler (v4.0.5) across the three Gene Ontology (GO) categories BP, CC and MF [84]. Only categories with FDR ≤ 10% were considered significantly enriched. Furthermore, hub genes within each module were identified.

### 4.7. Target Gene Selection for Validation by RT-qPCR

We selected lncRNAs considered as hub genes belonging to modules significantly correlated with one or both *Bothrops* venoms incubations in male and female worms separately, and that were DE in the male RNA-Seq results (FDR ≤ 5%). Among these, we selected lncRNAs that had (i) TPM > 1, (ii) fold-change (FC) > 2 in the RNA-Seq, and (iii) primer pairs designed between two exons. All selected lncRNAs also had evidence of expression in other *S. mansoni* life-cycle stages (Supplementary Figure S3) and were detected in single-cell RNA-Seq analysis (Supplementary Figure S5). As a control, *SmLINC143821-IBu* was selected as an lncRNA non-DE in the RNA-Seq analysis (Supplementary Figure S2).

Additionally, the five most stable non-DE protein-coding genes across all RNA-Seq data were chosen as candidate reference genes for the RT-qPCR assays, given the better suitability of condition-specific reference genes as normalizers over standard housekeeping genes [59,85]. NormFinder [86] and geNorm [87] software were employed, via the online tool RefFinder [88] (https://www.ciidirsinaloa.com.mx/RefFinder-master/, accessed on 2 February 2026), to determine the two most stable candidate reference genes, and *Smp_136320.1* and *Smp_336360.1* were used to normalize the Cycle threshold (Ct) values and relative expression. RefFinder results are shown in Supplementary Table S14.

### 4.8. Primer Design and Validation by RT-qPCR

All primer pairs for DE lncRNAs and candidate reference genes were designed using Primer3 (https://bioinfo.ut.ee, accessed on 10 October 2025) and between two exons of selected genes. The primers were designed following the parameters: maximum primer size of 25 nt, annealing temperature ranging from 57 °C to 63 °C, GC content between 30% and 60%, product size varying between 90 and 250 nt, and a maximum allowable length of a mononucleotide repeat set to three. All primers were purchased from Thermo Fisher. Primer’s efficiencies were tested through RT-qPCR using 5 µL of 1× LightCycler 480 SYBR Green I Master Mix (Roche Diagnostics, Basel, Switzerland), 800 nM of each primer and 2.5 µL of 2-fold serial diluted cDNA samples obtained from a pool of *S. mansoni* male and female samples on a LightCycler 480 System instrument (Roche Diagnostics). The primer sequences and efficiencies are shown in Supplementary Table S15. RT-qPCR was performed using cDNA samples of males (three biological replicates) incubated with *B. jararacussu* or *B. moojeni* venoms and negative controls. The RT-qPCR assays were performed using 5 µL of 1× LightCycler 480 SYBR Green I Master Mix (Roche Diagnostics, Basel, Switzerland) with 800 nM of each primer pair and 2.5 µL of samples on a LightCycler 480 System instrument (Roche Diagnostics). All primers were tested in technical triplicates for all samples, in addition to a no-template control (NTC). Raw Cts values are provided in Supplementary Table S16.

### 4.9. Statistical Analysis

For phenotypical results, one-way ANOVA with Dunnett’s post-hoc correction was performed. To calculate differential gene expression, the delta-Ct method was applied [89], considering the efficiency values of each primer pair. Normal distribution and homogeneity of variances were evaluated employing the Shapiro-Wilk and Levene tests, respectively. As all data passed these tests, differences in the RT-qPCR expression of the assessed genes between control and incubated samples were evaluated by one-tailed Student’s *t*-test assuming equal variances; of note, the RNA-Seq results, obtained with an experimental design identical to that used for the RT-qPCR sampling, indicated the expected direction of modulation of the target lncRNAs following venom incubation, justifying the use of a one-tailed test. Statistical analysis and graphical representation were performed with R 4.5.2 and GraphPad Prism 8.4.3.

## Data Availability

Sequencing data were deposited at the NCBI Sequence Read Archive (SRA) under the BioProject accession number PRJNA1442209. The original contributions presented in this study are included in the article/Supplementary Materials. Further inquiries can be directed to the corresponding author.

## Supplementary Materials

The following supporting information can be downloaded at: https://www.mdpi.com/article/10.3390/ijms27115027/s1.

## Abbreviations

The following abbreviations are used in this manuscript:

5-AzaC	5-Azacytidine
BP	Biological Process
CC	Cellular Component
Ct	Cycle threshold
CV	Coefficient of variation
DE	Differentially expressed
DEGs	Differentially expressed genes
FC	Fold change
FDR	False discovery rate
GO	Gene Ontology
kME	Module eigengene-based connectivity
lncRNA	Long non-coding RNA
lincRNA	Long intergenic non-coding RNA
lncaRNA	Long antisense non-coding RNA
lncsRNA	Long sense non-coding RNA
MF	Molecular Function
PZQ	Praziquantel
RNA-Seq	RNA Sequencing
RSEM	RNA-Seq by Expectation Maximization
SEM	Standard error of the mean
STAR	Spliced Transcripts Alignment to a Reference
TPM	Transcripts per million
TOM	Topological Overlap Matrix
VST	Variance stabilizing transformation
WGCNA	Weighted Gene Co-expression Network Analysis
