# Supplementary material for: Long Non-Coding RNAs Identified as Hub Genes by Weighted Gene Co-Expression Network Analysis in Schistosoma mansoni Following Incubation with Bothrops Snake Venoms
Source: Int J Mol Sci. 2026 Jun 2;27(11):5027. doi: 10.3390/ijms27115027 (PMC13256390; doi:10.3390/ijms27115027)
Supplement: Supplementary file 1 [file ijms-27-05027-s001.zip › Supp-Material-ijms-27-05027-03jun26.pdf]

International Journal of *Molecular Sciences*

MDPI

Article - Supplementary Material

Long non-coding RNAs identified as hub genes by Weighted Gene Co-Expression Network Analysis in *Schistosoma mansoni* following incubation with *Bothrops* snake venoms

Marina Zenga-Carrenho[1†], Agatha Fischer-Carvalho[1†], Tereza Cristina Taveira-Barbosa[1], Pedro Jardim Poli[1], Vilaça Guimarães-Oliveira[1,2], Alison Felipe Alencar Chaves[3], Solange M. T. Serrano[3], Ana Carolina Tahira[1], Sergio Verjovski-Almeida[1,4], Murilo Sena Amaral[1*]

[1] Laboratório de Ciclo Celular, Instituto Butantan, São Paulo 05503-900, Brazil
[2] Programa de Pós-Graduação Interunidades em Biotecnologia, Universidade de São Paulo, São Paulo 05508-000, Brazil
[3] Laboratório de Toxinologia Aplicada, Center of Toxins, Immune-Response, and Cell Signaling (CeTICS), Butantan Institute, São Paulo 05503-900, Brazil
[4] Departamento de Bioquímica, Instituto de Química, Universidade de São Paulo, São Paulo 05508-000, Brazil
* Correspondence: murilo.amaral@butantan.gov.br
† These authors contributed equally to this work

Supplementary Material

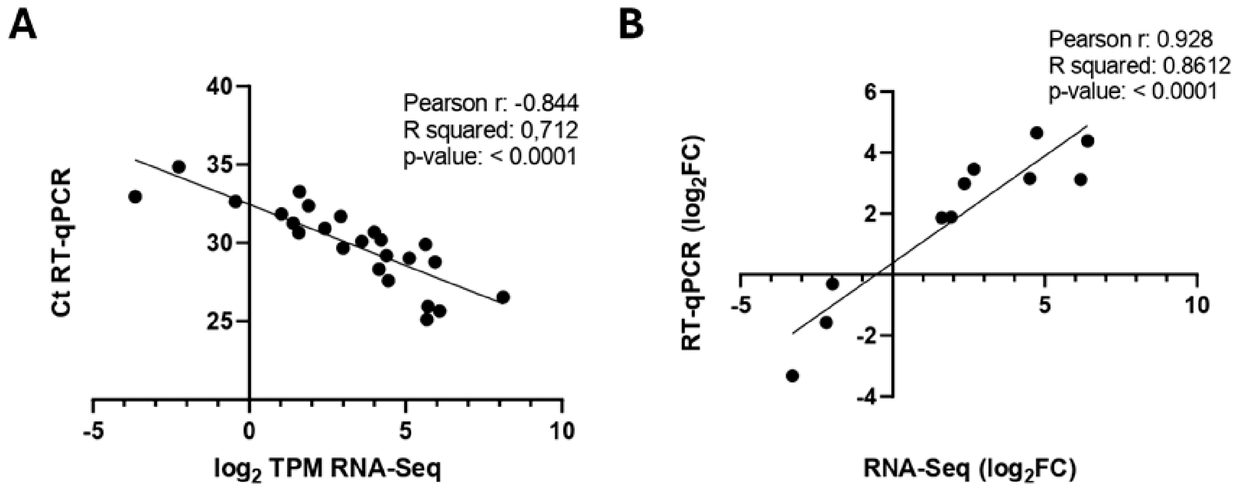

Supplementary Figure S1: Correlations between RNA-Seq and RT-qPCR data. (**A**) Correlation between genes raw Cycle threshold (Ct) in the RT-qPCR validation and $\log_2$ transcripts per million (TPM) in the RNA-Seq. (**B**) Correlation between genes $\log_2$ fold-change (FC) in the RT-qPCR validation and $\log_2$ FC in the RNA-Seq.

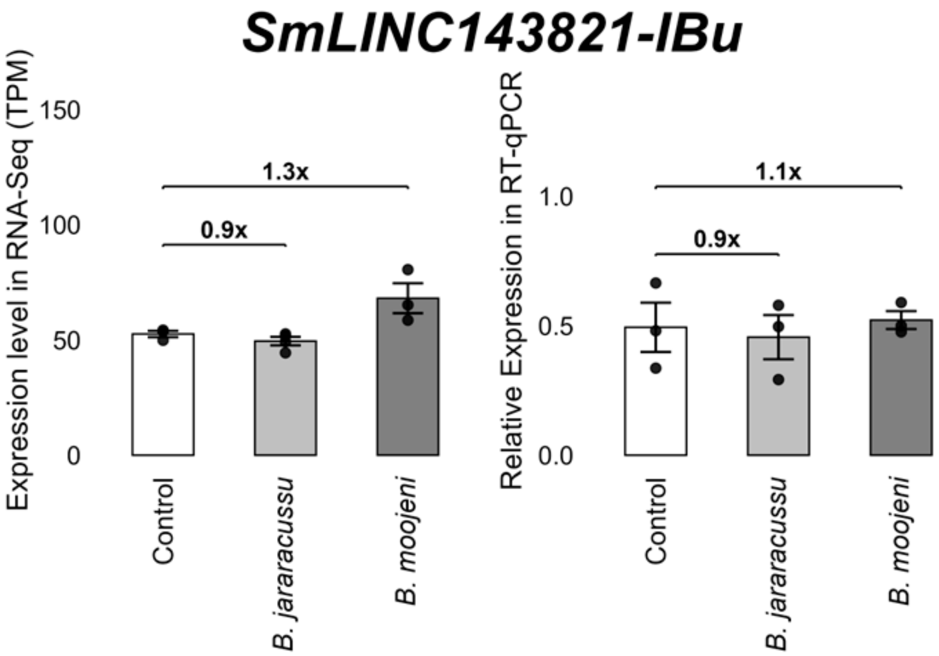

Supplementary Figure S2: Expression levels in RNA-Seq (expressed in TPM, transcripts per million) (left) and gene expression in RT-qPCR (right) of *SmLINC143821-IBu*, a lncRNA non-differentially expressed in male *Schistosoma mansoni* samples incubated with *Bothrops jararacussu* or *Bothrops moojeni* venoms. Relative gene expression in RT-qPCR was calculated using the delta Ct approach considering each pair of primers efficiency and normalizing to the average of two selected reference genes (*Smp_136320.1* and *Smp_336360.1*). Means ± SEM of three biological replicates were used. Numbers above horizontal lines between bars indicate the fold-changes (FCs), calculated by dividing the treatments' gene expression by control´s gene expression in both RNA-Seq and RT-qPCR results. A Student's t test between incubated samples groups and their respective control groups was applied assuming equal variances. No significant changes were detected.

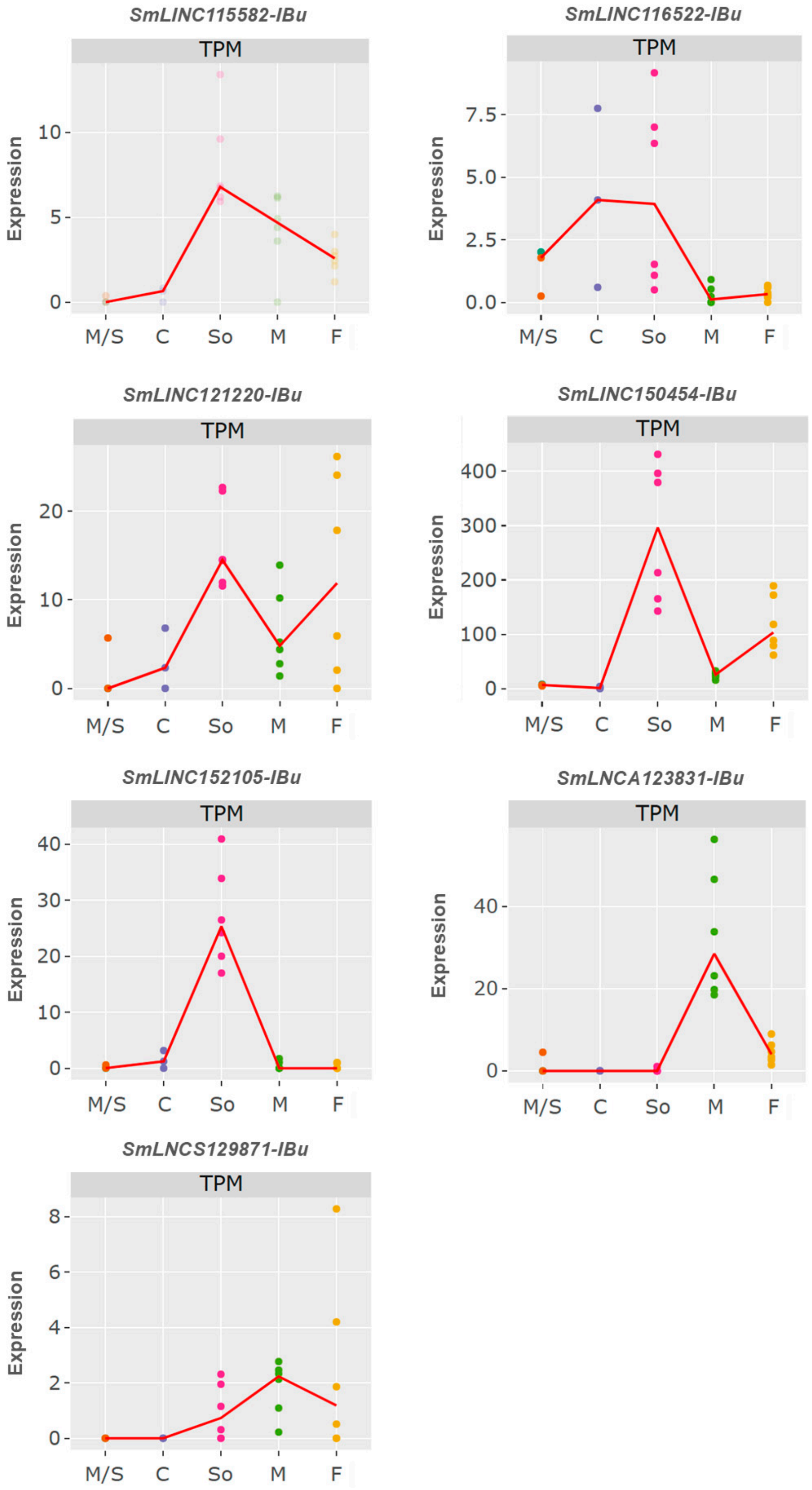

Supplementary Figure S3: **Expression levels (expressed in TPM – transcripts per million) of seven long non-coding RNAs (lncRNAs) selected for validation by RT-qPCR across six life-cycle stages of *S. mansoni*.** This data is available online at https://verjolab.shinyapps.io/Reference-genes. M/S = Miracidia/Sporocysts. C = Cercariae. S = Schistosomula. M = Males. F = Females.

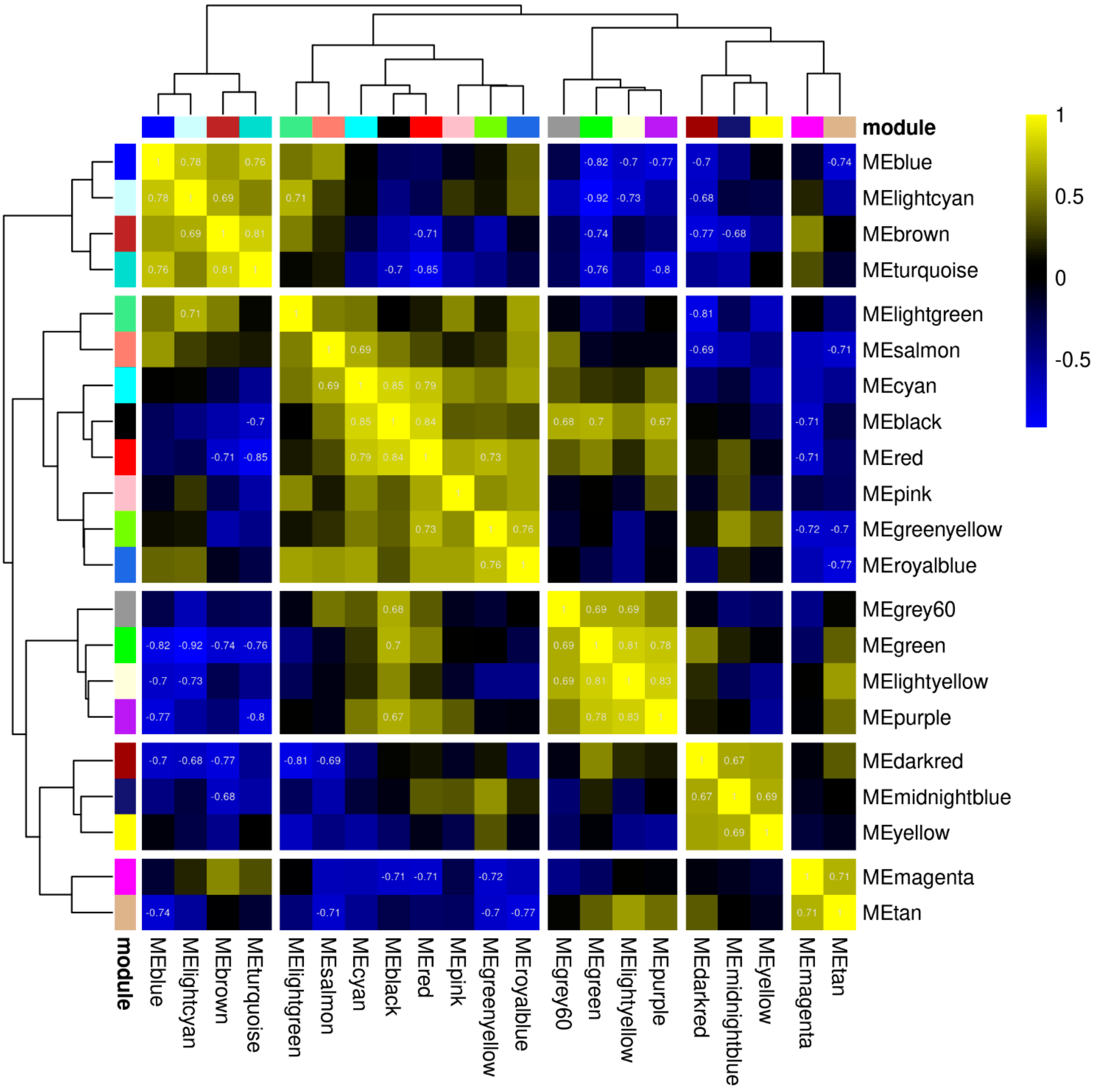

Supplementary Figure S4: Correlation between modules obtained in Weighted Gene Co-Expression Network Analysis for male *Schistosoma mansoni* samples incubated with *B. jararacussu* or *B. moojeni* venoms. Only the values of significant Pearson's correlations ($p < 0.05$) are shown. The eigengene (ME) values of each module were used to build the correlation matrix. The scale color represents high negative (blue) to high positive (yellow) correlations, those without meaningful values are colored in black tones.

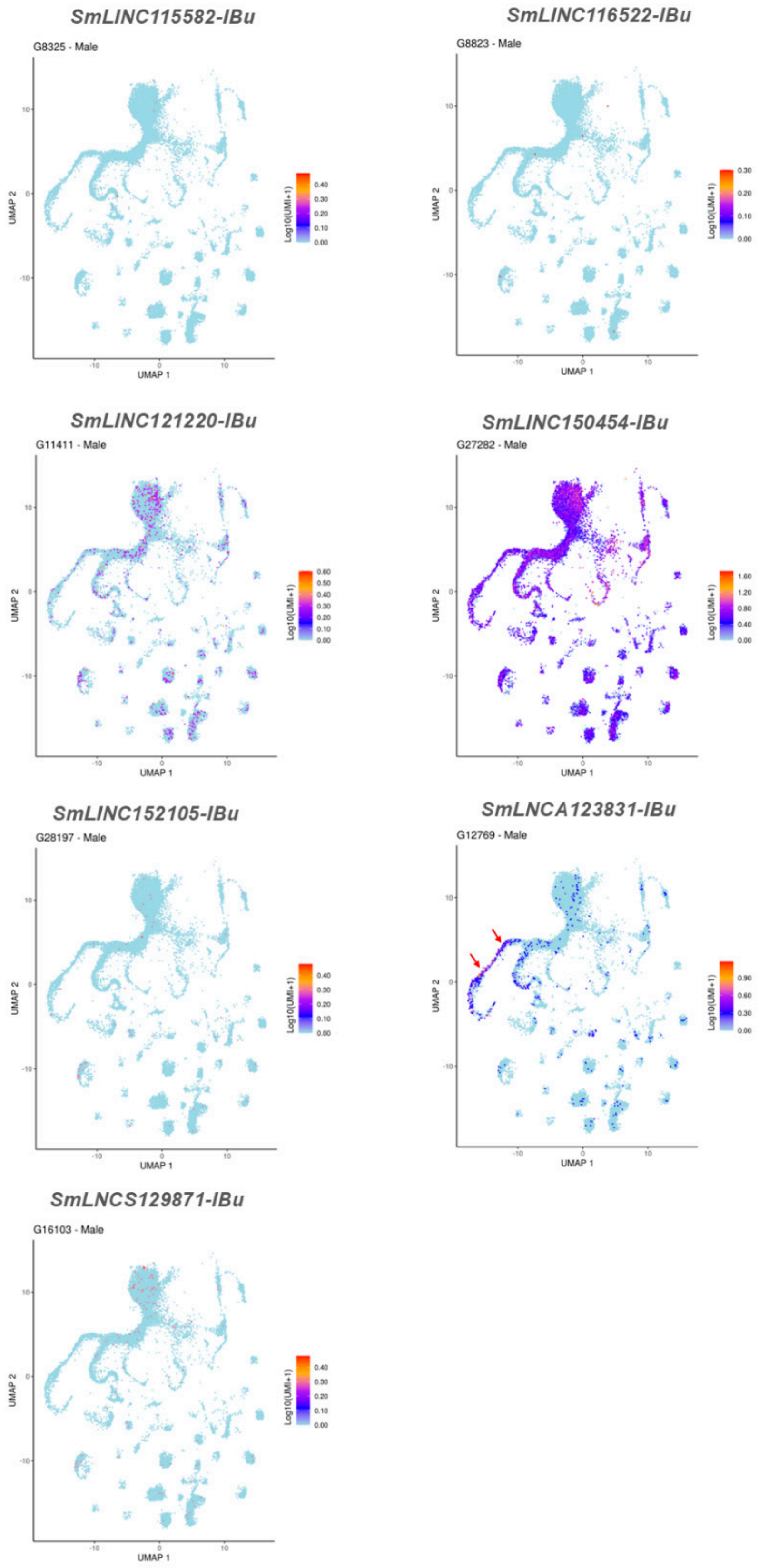

Supplementary Figure S5: Single-cell clusters expression profiles of the seven lncRNAs selected for validation by RT-qPCR on *S. mansoni* adult worms. UMAP plots show the expression enrichment of genes and are colored by gene expression (blue = low, red = high). The scale represents $\log_{10}$ (UMIs+1). Red arrows indicate *SmLNCA123831-IBu* enrichment in *meg1*+ and *egc1*+ single-cell clusters. Single-cell expression data is available online at http://verjolab.usp.br:8081/cluster-search/.

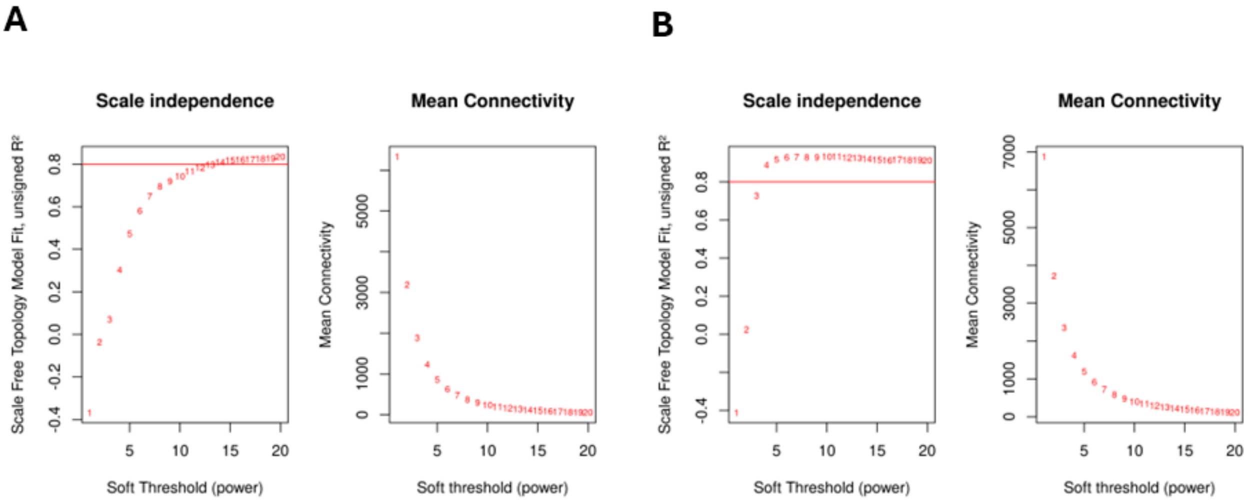

Supplementary Figure S6: Scale-free topology plots according to the soft-thresholding power for female (A) and male (B) samples. The left panel shows the scale-free network topology fit index as a function of increasing power. The y-axis represents the R^2 value, and the x-axis represents the power used. The red line indicates $R^2 = 0.8$, which corresponds to the minimum threshold considered for approximating a scale-free topology. The right panel shows the mean connectivity of the networks as a function of increasing power. The y-axis represents the mean connectivity values, and the x-axis shows the power values.

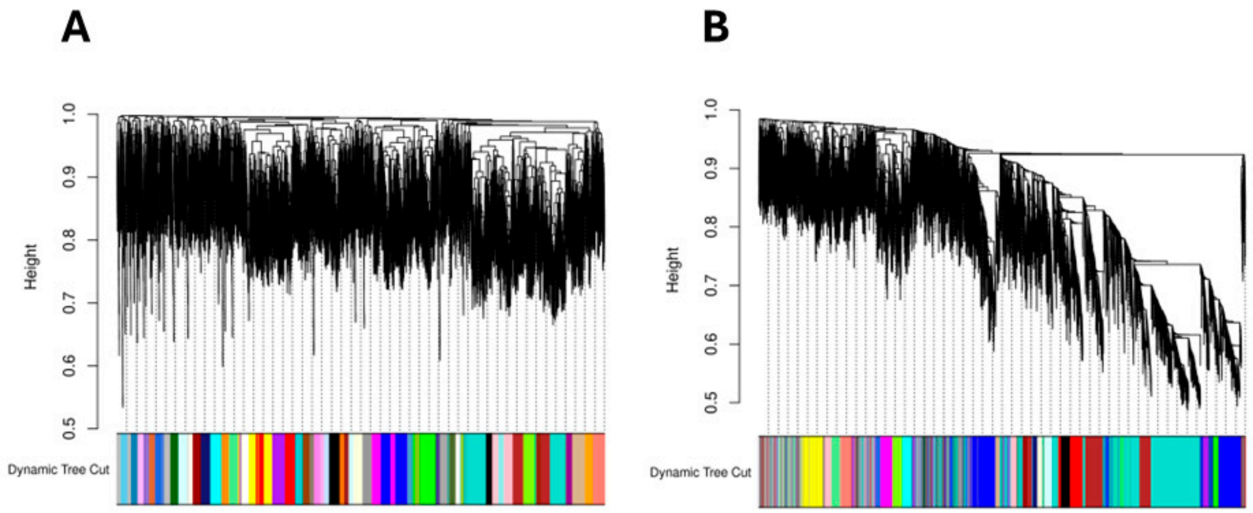

Supplementary Figure S7: Dendrogram of modules identified through Weighted Gene Co-expression Network Analysis (WGCNA) using female (A) and male (B) samples incubated with *Bothrops jararacussu* or *Bothrops moojeni* venoms or incubated with PBS (controls). A total of 49 modules were identified in female samples and 21 in male samples. Genes are represented as branches in the upper part of the dendrogram, and the identified modules are shown as colored bars directly below. The y-axis (height) represents the distance, calculated using the dissimilarity measure (1 – TOM).

Disclaimer/Publisher's Note: The statements, opinions and data contained in all publications are solely those of the individual author(s) and contributor(s) and not of MDPI and/or the editor(s). MDPI and/or the editor(s) disclaim responsibility for any injury to people or property resulting from any ideas, methods, instructions or products referred to in the content.
